# Afocal Optical Flow Sensor for Reducing Vertical Height Sensitivity in Indoor Robot Localization and Navigation

**DOI:** 10.3390/s150511208

**Published:** 2015-05-13

**Authors:** Dong-Hoon Yi, Tae-Jae Lee, Dong-Il “Dan” Cho

**Affiliations:** 1 Department of Electrical and Computer Engineering, Automation and Systems Research Institute (ASRI), Seoul National University, Seoul 151-742, Korea; E-Mails: ydh01@snu.ac.kr (D.-H.Y.); ltj88@snu.ac.kr (T.-J.L.); 2 Inter-University Semiconductor Research Center (ISRC), Seoul National University, Seoul 151-742, Korea

**Keywords:** optical flow sensor, indoor robot localization, navigation, dead reckoning

## Abstract

This paper introduces a novel afocal optical flow sensor (OFS) system for odometry estimation in indoor robotic navigation. The OFS used in computer optical mouse has been adopted for mobile robots because it is not affected by wheel slippage. Vertical height variance is thought to be a dominant factor in systematic error when estimating moving distances in mobile robots driving on uneven surfaces. We propose an approach to mitigate this error by using an afocal (infinite effective focal length) system. We conducted experiments in a linear guide on carpet and three other materials with varying sensor heights from 30 to 50 mm and a moving distance of 80 cm. The same experiments were repeated 10 times. For the proposed afocal OFS module, a 1 mm change in sensor height induces a 0.1% systematic error; for comparison, the error for a conventional fixed-focal-length OFS module is 14.7%. Finally, the proposed afocal OFS module was installed on a mobile robot and tested 10 times on a carpet for distances of 1 m. The average distance estimation error and standard deviation are 0.02% and 17.6%, respectively, whereas those for a conventional OFS module are 4.09% and 25.7%, respectively.

## 1. Introduction

Mobile robots are widely applied in indoor environments for various purposes, including floor cleaning, guidance, and surveillance. Accurate localization allows the mobile robots to accurately perform mapping, obstacle avoidance, and path planning [1]. Localization can be further decomposed into two types: absolute and relative [2]. Absolute localization relies on landmarks, maps, beacons, or satellite signals to determine the global position and orientation of the robot. Relative localization is usually used in the process model of estimators for absolute localization, or when absolute localization sensor measurements such as GPS are denied for a moment [3,4,5,6].

Dead reckoning (DR) is commonly used for the intermediate estimation of position during movement. DR is often used when wheel encoders are available for drive wheel position measurement. Encoders are inexpensive and allow very high sampling rates; however, because of the errors in kinematic model parameters, wheel slippage, or other subtle causes, poor position estimates may occur. Poor estimates in position during path execution require more frequent path re-planning, incurring extra overhead, and possibly slowing the time required to complete the mission. Therefore, it is important to minimize errors in estimated position during the path execution phase. 

There has been interest in the use of optical flow techniques for vision-based mobile robot localization and navigation. McCarthy and Bames [7] presented a comparison of four optical flow methods and temporal filters for mobile robot navigation. The strongest performances were achieved using Lucas and Kanade and a recursive filter. Campbell *et al.* [8] proposed criteria and an experimental method for evaluating optical flow for visual odometry systems in real, unstructured environments. The optical flow was used to aid in the navigation of an omnidirectional robot [9]. A charge-coupled devices camera was positioned at a 45° downward angle with respect to the ground in front of the robot. The obtained optical flow was combined with the DR results using the maximum likelihood technique. The method used to calculate optical flow is quite complex and requires a large number of computations to obtain good results.

Using the two-dimensional displacement output from optical mouse sensors, which are small, inexpensive, non-contact devices have been suggested as an alternative method of odometry estimation [10,11,12,13,14,15,16,17,18,19]. Optical mouse sensors integrate a small complementary metal oxide semiconductor camera (of the order of 30 × 30 pixels) with digital signal processing hardware and proprietary firmware algorithms to infer the displacement in both the X and Y directions based on the optical flow of features identified in consecutive image frames [20]. The use of optical mouse sensors for odometry has clear advantages over using wheel encoders; specifically, the independence with respect to kinematic forces such as wheel slippage and movement are resolved in more than one axis, which is of particular interest for omnidirectional platforms [21].

Several sources of error can be identified in the use of optical mouse sensors, including surface type, height variance, lighting conditions, and the angular displacement [13,17]. Vertical height variance is a key source of odometry error when using optical mouse sensors [11,13,22]. The errors induced by height variance are related to the scaled distance inferred by the optical flow algorithm. The larger surface area represented by each pixel causes the sensor to read successively lower values. Hence, the highest count values sampled are closest to the floor, where each pixel occupies the minimum area and thus appears to move the greatest distance.

Generally, vertical height variance is a result of the robot moving over irregular surfaces; this is the dominant cause of systematic error in horizontal displacement measurements. Researchers have characterized error variances for different surfaces, particularly those commonly used in indoor flooring [13,21]. The results demonstrated that different ground surfaces yield different sensitivities, with variances typically less than 10%. Larger variances from the mean are typically recorded on rougher surfaces (e.g., carpet or stoneware) or over significant discontinuities (e.g., joins between tiles), suggesting that the variances are related to the previously identified vertical variance errors.

To minimize error induced by height variance, many of the existing implementations use a surface with minimal height variance and mount the optical mouse sensor assembly (including the lens) in direct contact with the surface being measured [11,12,13]. The use of contact-free refocused optical mouse sensors for odometry estimation has been investigated in [23,24,25,26], and the results show the sensor data is linear with respect to height [24]. Scarab, the lunar rover prototype developed at Carnegie Mellon University, uses four commodity optical mouse sensors, each of which is attached to a lens of a different focal length [27]. A differential optical navigation sensor for mobile robots that provides consistent output despite changes in the distance between the surface and the sensor has also been proposed using two OFSs [28].

In this paper, we aim to mitigate the systematic error induced by height variance in indoor mobile robots using an afocal optical mouse sensor module that consists of single optical mouse sensor, a refocused lens, and a pinhole. Using the methods described in this paper, accurate estimates of position can be maintained even when wheel slip occurs or uneven ground is encountered.

## 2. Methods

### 2.1. Afocal Optical Flow Sensor System 

**Figure 1:**
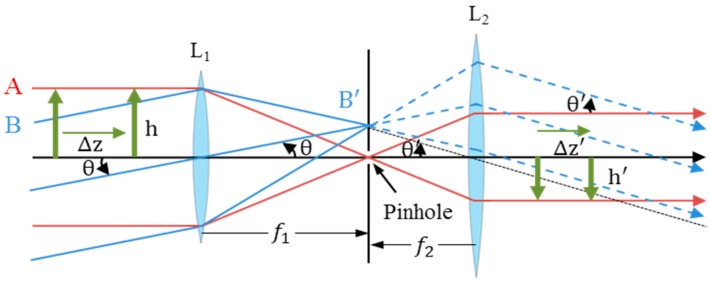
Diagram of the afocal system.

To mitigate errors induced by height variance, we propose an afocal OFS system with a pinhole located at the near focus of the first lens from an OFS. An afocal system is an optical system that produces no net convergence or divergence of the beam (*i.e.*, it has an infinite effective focal length). An afocal system is formed by the combination of two focal systems. The near focal point of the first system is coincident with the front focal point of the second system. The rays parallel to the horizontal axis in object space are conjugate to the rays parallel to the axis in image space. Common afocal systems include a telescope imaging a star (the light entering the system is at infinity, and the image it forms is at infinity), binoculars, and beam expanders [29]. Figure 1 shows an afocal system in which the “A” rays parallel to the axis are only passed through the pinhole, whereas the “B” rays are not parallel to the axis, which would be blocked by the pinhole screen. In Equation (1), longitudinal magnification is constant and determined only by the focal lengths of the two lenses.

(1) $$m=\frac{f_{2}}{f_{1}}=\frac{h'}{h}$$

where *m*, $f_{1}$, $f_{2}$, h, and h' are the longitudinal magnification, the near focal length of lens L_1_, the front focal length of lens L_2_, the subject, and the conjugated scale-changed subject, respectively.

**Figure 2:**
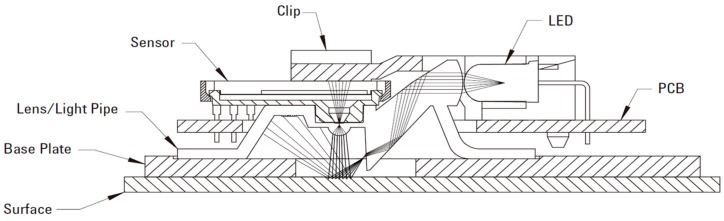
Cross-sectional view of the printed circuit board assembly components for the OFS ADNS-3080 (courtesy of Avago).

The OFS selected for this work is the ADNS-3080 (Figure 2) [20], a common OFS that includes an internal low-resolution camera and a digital signal processor (DSP) programmed to estimate the relative displacement of the micro-shadows of the acquired images [30]. An OFS estimates a moving distance by analyzing with the reflected images from the active illuminated surface. If there are no irregularities on the surface, or if the surface is not sufficiently reflective, like glass, it cannot be used properly. This family of optical sensors uses complementary metal oxide semiconductor technology that integrates the camera and the DSP into the same chip. The sensor computes the optical flow by performing a comparative analysis of the sequence of images acquired of a flat surface in front of the sensor in order to estimate the motion of the surface [31]. The motion can be measured at different resolutions such as 800 counts per inch. Under this specific configuration, the OFS is internally calibrated to measure a pulse when a plain surface in front of the sensor is translated by 31.75 μm. Any change in the height between the sensor and the surface requires the development of a calibration procedure to convert the counts measured by the sensor to an estimate of the physical magnitude under measurement [32]. Palacin *et al.* [13] introduce an optical mouse calibration method using a MATLAB function.

The original design of an optical mouse based on this optical sensor combines three different parts: an OFS; an external light emitting diode (LED) to illuminate the surface in front of the sensor; and a small plastic structure that includes two convex lenses to focus the light of the LED and the image acquired by the OFS. The combination of all these parts was optimized to acquire focused images of a surface at a very low height (from 2.3 mm to 2.5 mm); although, this range can be increased by replacing the originally provided lens.

The ADNS-3080 includes an internal imaging device with a sensitive 30 × 30 array of gray intensity pixels. This OFS operates at very high frame rates and has a very fast internal shutter. It was originally designed to acquire images of a flat surface (approximately 1.82 mm × 1.82 mm) at a very low height (2.4 mm) to estimate displacement within images. The ADNS-3080 has a standard serial peripheral interface bus to read/write the internal registers and control the common actions of the sensor. According to the manufacturer’s specifications, the optical sensor can acquire and process images at a very fast speed (from 2000 to 6469 frames per second) due to the low sizes of the images acquired (30 × 30 pixels) [26].

**Figure 3:**
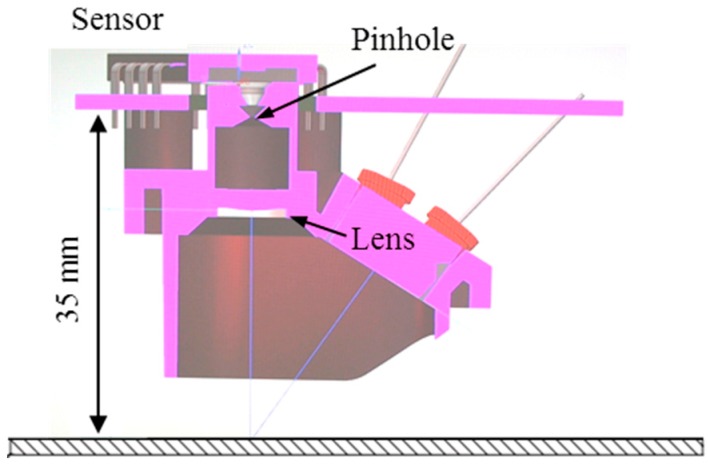
Cross-sectional view of the a focal OFS module with a pinhole diameter of 0.5 mm and a lens with a focal length of 10 mm, which images the floor onto the light-sensitive area of the sensor.

The newly designed afocal OFS module consists of a pinhole with a diameter of 0.5 mm, a lens with a focal length of 10 mm (L1 in Figure 1), and an ADNS-3080 OFS that replaces the second lens in the afocal system (see Figure 3). As the pinhole sizes gets bigger, more less-columnated light rays enter through the pinhole, which makes the image less clear, which in turn increases the sensitivity to height variations. Note, that if the pinhole size is too small, there is insufficient light for the afocal OFS to recognize the surface pattern. We have selected a pinhole diameter of 0.5 mm with 4 LEDs in this study. The smaller pinhole size gives clear images and blocks more nonparallel light, whereas the sensor needs more light in order to obtain brighter images [33]. 

**Figure 4:**
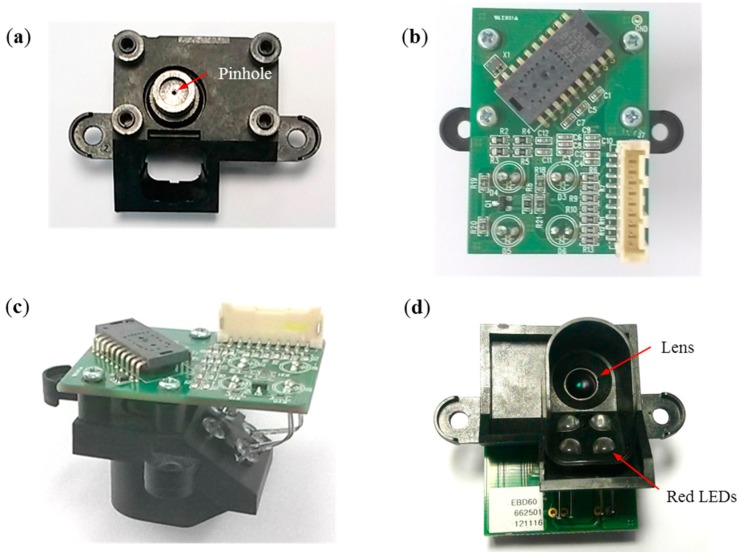
Fabricated afocal OFS module: (**a**) top view without circuit board; (**b**) top view with circuit board; (**c**) perspective view; and (**d**) bottom view.

The fabricated afocal OFS module at different angles is shown in Figure 4. Figure 4a depicts the pinhole, Figure 4b shows the ADNS-3080 optical mouse chip, Figure 4c shows the perspective view of the afocal OFS module, and Figure 4d shows the lens and the red LEDs.

### 2.2. Experimental Setup 

**Figure 5:**
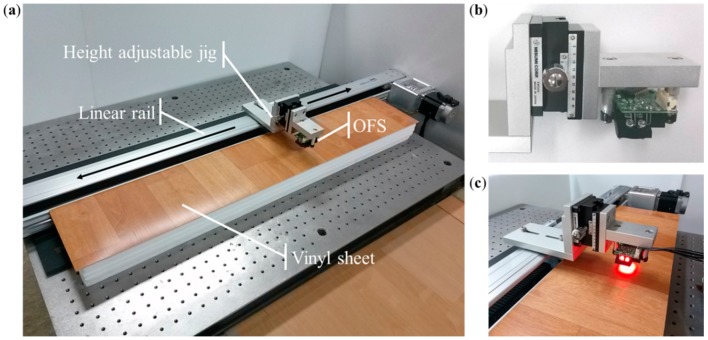
Experimental setup: (**a**) robotic gantry system; (**b**) Misumi XWG60 rack-and-pinion-type linear stage positioner; and (**c**) activation image of the afocal OFS module.

To obtain accurate and repeatable data, a robotic gantry system consisting of a height-adjustable jig and a belt-driven linear actuator is used (Figure 5). The linear actuator (DRSB99SL40-ST1000, Dream Robot System, Incheon, Korea) is capable of a repeat precision of 20 μm, an accuracy of 50 μm, and a maximum speed of 1000 mm/s [34]. Because of the 100 cm maximum moving distances of the linear actuator, the range is set to 80 cm in the experiments. A rack and pinion-type linear stage positioner (XWG60, Misumi, Tokyo, Japan) is used to precisely adjust the height [35].

A microcontroller unit communicates with the sensor via one serial peripheral interface channel. A Microchip processor (dsPIC33FJ256) employing a powerful 16-bit architecture that seamlessly integrates the control features with the computational capabilities of a digital signal processor is used [36]. The sampling time to obtain the motion information from the sensor is 20 ms.

**Figure 6:**
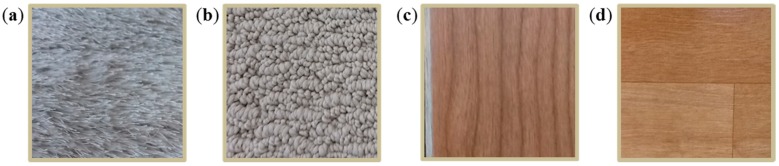
Floor materials used in this work: (**a**) texture-style carpet; (**b**) loop-style carpet; (**c**) laminate floor; and (**d**) vinyl sheet.

Most of the causes of height variation in indoor navigation, excluding variations associated with robot suspension, are rough carpet, doorsills, low obstacles, uneven ground, and any objects that a robot can climb. To verify the feasibility of the fabricated afocal OFS module in these environments, four types of floor materials were selected for experiments: laminated floor; vinyl sheet; texture-style carpet; and loop-style carpet (Figure 6). The height between the OFS and the surface of the material is varied from 30 to 50 mm in seven steps, and the sensor module is moved by the robotic gantry system over a distance of 80 cm at a speed of 35 cm/s. The same experiments are repeated 10 times, and the image quality is examined every 5 mm in the experimental height interval.

**Figure 7:**
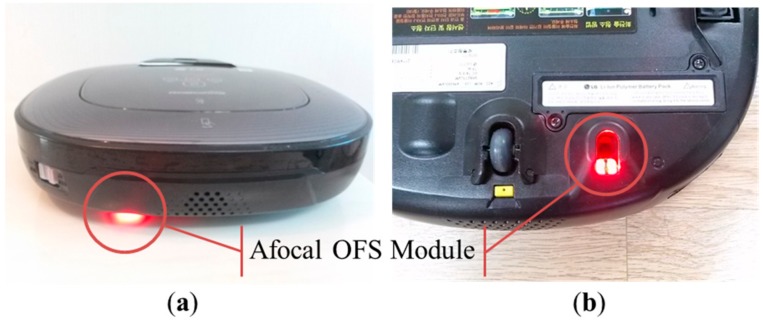
Vacuum-cleaning robot equipped with an afocal OFS module: (**a**) rear view and (**b**) bottom view.

**Figure 8:**
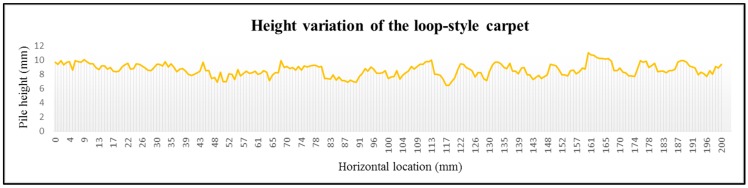
Laser measured surface profile of the loop-style carpet.

Finally, the afocal OFS module is installed on a mobile robot (Figure 7) and tested 10 times on the loop-style carpet (Figure 6b) over distances of 1 m. Since the surface height variation of the loop-style carpet is the greatest among the other flooring materials (Figure 6), the loop-style carpet is selected for robot experiments. The height variation of the loop-style carpet is shown in Figure 8. The surface profile is measured by a laser distance sensor (DLS-B 30, DIMETIX, Herisau, Switzerland) [37], which has a resolution of 0.1 mm and the robotic gantry system. The laser sensor is moved from 0 to 200 mm by 1 mm intervals with the linear motion guide, and the surface profile is measured. The average height and SD are 86 mm and 8.7 mm, respectively. 

Moving distance information is gathered while the robot is moved by manual operation. After the acquisition of the experimental data, the average moving distance estimation error and SD are calculated.

## 3. Results and Discussion 

**Figure 9:**
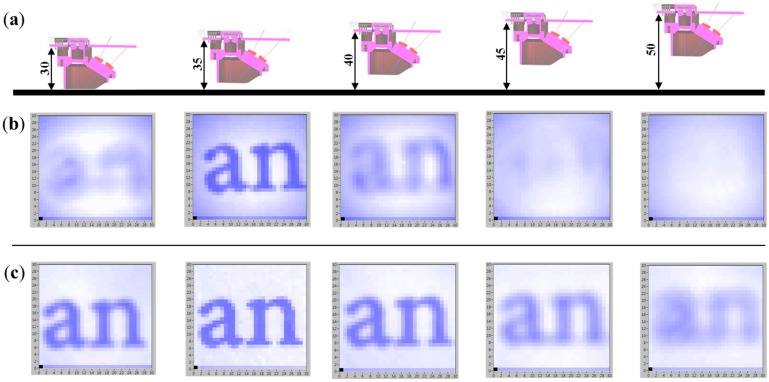
Image acquisition for different OFS module at different heights: (**a**) sensor height in mm; (**b**) images obtained using the conventional fixed-focal-length OFS module; and (**c**) images obtained using the proposed afocal OFS module.

This section analyzes the performance of the suggested afocal OFS module in robustly estimating distance in indoor environments with height variation. A first experiment was carried out to determine if the use of the suggested sensor system improved image clarity. The images of the word “an” (MS Word, 9 pt. font size, Times New Roman) obtained with the OFS module are shown in Figure 9. The images are clearer compared to those obtained without a pinhole, as the height changes between the sensor and the test floor from 30 mm to 50 mm at intervals of 5 mm. The only difference between the afocal OFS module and conventional fixed-focal-length OFS module used in this test is the existence of the pinhole. The conventional OFS module is calibrated at the height of 35 mm. Since the conventional OFS module does not have pinhole, it can be regarded as having a big pinhole. As the pinhole size becomes bigger, more less-columnated light rays enter through the pinhole, which makes the image less clear, which in turn increases the sensitivity to height variations.

**Figure 10:**
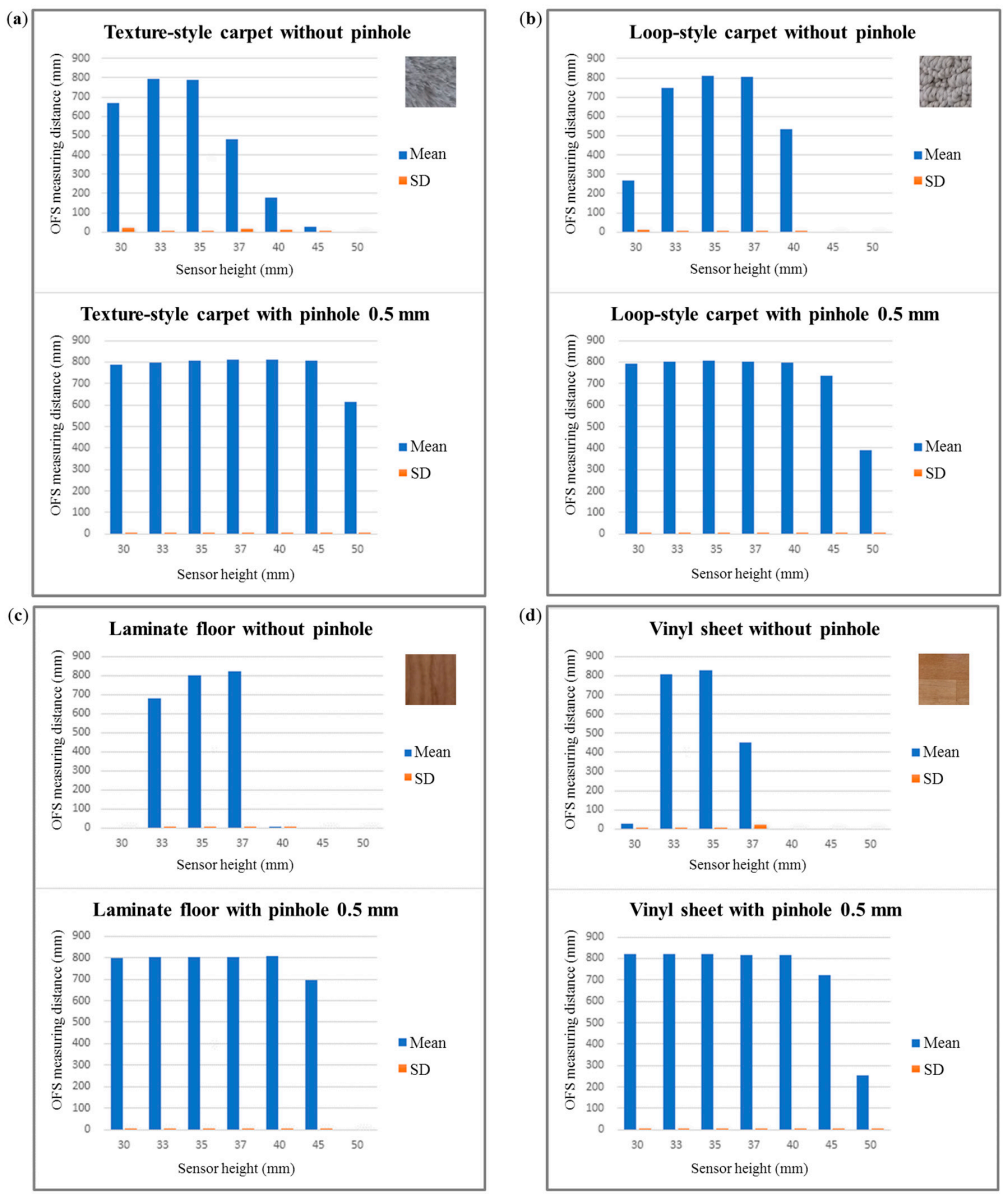
OFS-measured mean distances and standard deviations after 10 repeated measurements on each floor type at different heights: (**a**) texture-style carpet; (**b**) loop-style carpet; (**c**) laminate floor; and (**d**) vinyl sheet.

The measured mean distance and SD are shown in Figure 10. In addition to the data in Figure 10, Table 1 includes the error percentages obtained using the afocal OFS module while varying the height between the afocal OFS module and the floor material. The results show that a 1-mm offset in sensor height induces a 0.1% systematic error in the height range of 30 mm to 40 mm; in comparison, the systematic error for a 1 mm offset in a conventional OFS module with a fixed focal length in the height range of 30 mm to 35 mm is 14.7%. In Table 1, the results show that when the height of the afocal OFS sensor rises over 40 mm the error rates start to increase. This is because the pinhole size is not ideally zero, and the illumination on the surface decreases as the height increases.

**Figure 11:**
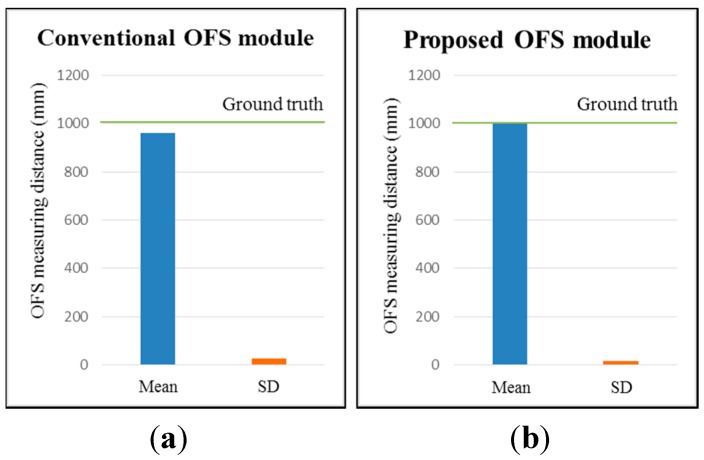
OFS-measured mean distances and standard deviations after 10 repeats on the loop-style carpet in a mobile robot: (**a**) conventional OFS module and (**b**) proposed afocal OFS module.

The test results for a mobile robot with an installed OFS module are shown in Figure 11 and Table 2. For ten 1000 mm-movements of the robot over loop-style carpet, the average estimated distance is 1000.2 mm (0.02% distance error), and the SD is 17.6. For the conventional OFS module with a fixed focal length, the average estimated distance is 959.1 mm (4.09% distance error), and the SD is 25.7.

**Table 1:** Computed distance error for various module heights.

Floor Material	Height (mm)	Conventional Fixed-Focal-Length OFS Module			Proposed Afocal OFS Module		
		Mean (mm)	Standard Deviation	Error (%)	Mean (mm)	Standard Deviation	Error (%)
Laminate Floor	30	0	0	100.0	799	0.9	0.1
	33	678	2.9	15.3	802	0.9	0.2
	35	802	0.4	0.3	804	0.8	0.5
	37	823	0.7	2.9	805	0.7	0.6
	40	8	1.4	99.0	806	0.5	0.8
	45	0	0	100.0	694	2.9	13.3
	50	0	0	100.0	0	0.0	100.0
Vinyl Sheet	30	29	2.4	96.4	820	0.4	2.5
	33	809	2.2	1.1	820	0.5	2.5
	35	827	0.6	3.4	820	0.6	2.5
	37	452	22.1	43.5	819	0.7	2.3
	40	0	0	100.0	817	0.7	2.1
	45	0	0	100.0	722	2.4	9.8
	50	0	0	100.0	253	2.4	68.4
Texture-Style Carpet	30	671	20.6	16.1	789	2.6	1.4
	33	796	6.5	0.5	799	1.6	0.2
	35	790	6.8	1.3	808	0.6	1.0
	37	482	18.3	39.8	810	0.6	1.3
	40	179	9.1	77.6	812	0.5	1.5
	45	28	4.7	96.5	807	1.5	0.9
	50	0	0	100.0	614	4.4	23.3
Loop-Style Carpet	30	267	9.5	66.6	794	0.5	0.8
	33	749	2.3	6.4	802	0.4	0.2
	35	810	0.3	1.3	809	0.3	1.1
	37	803	1.6	0.4	804	0.5	0.4
	40	532	3.4	33.5	798	0.6	0.3
	45	0	0	100.0	737	1.8	7.9
	50	0	0	100.0	390	5.9	51.3

**Table 2:** Estimated moving distances for 1 m movements of mobile robots on loop-style carpet.

Conventional Fixed-Focal-Length OFS Module			Proposed Afocal OFS Module		
Mean (mm)	Standard Deviation	Error (%)	Mean (mm)	Standard Deviation	Error (%)
959.1	25.7	4.09	1000.2	17.6	0.02

## 4. Conclusions

In this paper, a novel method for more scale invariant afocal OFS module with single OFS in height varying indoor environments was introduced and experimented. To minimize the horizontal displacement measurement error due to OFS height variation while the robot is moving, an afocal system was applied to OFS for the first time. 

We conducted experiments to evaluate the performance of the proposed method in a linear motion guide on carpet, laminated floor, and vinyl sheet. The sensor height was varied from 30 mm to 50 mm in seven steps over a total distance of 80 cm. The experiments were repeated 10 times. The systematic error corresponding to a 1 mm change in sensor height for the proposed afocal OFS module was 0.1%; for comparison, the corresponding value for a conventional OFS module with a fixed focal length is 14.7%. Finally, the proposed afocal OFS module was installed on a mobile robot and tested 10 times on the loop-style carpet over distances of 1 m. The average distance estimation error and standard deviation (SD) for the new module were 0.02% and 17.6%, respectively; in contrast, the corresponding values for the conventional OFS module were 4.09% and 25.7%, respectively. As the afocal OFS moved away from the vertical height range of 30 mm to 45 mm, the measured moving distances decreased because the pinhole size was not ideally zero, and the illumination on the surface decreases as the height increases.

The proposed approach using an afocal system and an optical mouse sensor showed encouraging results and robust performance for accurate indoor odometry, even in the presence of localized high and low points. The afocal OFS could be an indispensable sensor for indoor robotic navigation.
